# Characterization of human cytomegalovirus infection dynamics in human microglia

**DOI:** 10.1099/jgv.0.002096

**Published:** 2025-04-29

**Authors:** Marcos Nuevalos Guaita, Tajudeen O. Jimoh, Emma B. Barrall, Kristina E. Atanasoff, Michelle E. Ehrlich, Sam Gandy, Estéfani García-Ríos, Pilar Perez Romero, J. Andrew Duty, Domenico Tortorella

**Affiliations:** 1 National Center for Microbiology, Instituto de Salud Carlos III. Majadahonda, Madrid, Spain; 2 Department of Microbiology, Icahn School of Medicine at Mount Sinai, New York, NY, USA; 3 Graduate School of Biomedical Sciences, Icahn School of Medicine at Mount Sinai, New York, NY, USA; 4 Department of Neurology, Icahn School of Medicine at Mount Sinai, New York, NY, USA; 5 Department of Genetics and Genomic Sciences, Icahn School of Medicine at Mount Sinai, New York, New York, USA; 6 Department of Pediatrics, Icahn School of Medicine at Mount Sinai, New York, New York, USA; 7 Department of Psychiatry and Alzheimer’s Disease Research Center, Icahn School of Medicine at Mount Sinai, New York, NY, USA; 8 James J Peters VA Medical Center, Bronx NY, USA; 9 Instituto de Agroquímica y Tecnología de Alimentos (IATA), Consejo Superior de Investigaciones Científicas (CSIC), Valencia, Spain; 10 Department of Biological Sciences, University of Notre Dame, Notre Dame, IN, 46556, USA; 11 Center for Therapeutic Antibody Development, Drug Discovery Institute, Icahn School of Medicine at Mount Sinai, New York, NY, USA

**Keywords:** antibodies, entry inhibitors, human cytomegalovirus, microglia, neuropilin-2 (NRP-2), tropism

## Abstract

Human cytomegalovirus (HCMV) is a β-herpesvirus that establishes asymptomatic infections in immunocompetent individuals but can cause severe or even life-threatening symptoms in immunocompromised patients. HCMV can replicate in a wide variety of cells through the engagement of diverse cell factors with the viral envelope protein gH/gL/gO (trimer) or gH/gL/UL128/UL130/UL131a (pentamer), allowing for systemic spread within the human host. This study explores HCMV infection tropism and dynamics in human microglia, demonstrating the susceptibility of microglia to both clinical and laboratory HCMV strains, *albeit* with lower efficacy for the laboratory strain, implying that both the gH/gL-trimer and -pentamer can mediate virus entry in microglia. The importance of the gH/gL pentamer for virus entry was demonstrated by the inhibition of virus infection upon pre-incubation with a soluble neuropilin-2 (NRP-2) entry factor. Further, we demonstrated that HCMV infection can be effectively inhibited by monoclonal antibodies specific for the gH/gL complexes and HCMV hyperimmunoglobulin. Lastly, we report that microglia infection can be prevented by newly characterized chemical entry inhibitors. Altogether, these findings underscore the potential of microglia as valuable models for studying HCMV neurotropism and strategies to block virus infection in cells that can impact neurological disorders.

**Impact Statement**

Human cytomegalovirus (HCMV) can infect diverse cell types, giving rise to numerous diseases including neurological diseases. The present study sought to investigate the viral tropism of brain-derived microglia cells. Our findings demonstrate that microglia are susceptible to HCMV infection of diverse strains, utilizing diverse virus envelope proteins for viral entry. Further, inhibitors that target the early steps of viral entry were effective at limiting infection. These studies provide the foundation to study HCMV tropism and replication in microglia.

## Introduction

Human cytomegalovirus (HCMV), also known as human herpesvirus 5, is a β-herpesvirus with broad infection capability. Although HCMV establishes benign infections in immunocompetent hosts, in individuals with immature or dysfunctional immune systems, HCMV is a major cause of morbidity and mortality [1]. HCMV entry into human cells occurs through either direct fusion or an endocytic pathway [1]. The viral attachment process involves specific interactions between viral anti-receptors (gH/gL/pUL128L and gH/gL/gO complexes) and cellular entry factors (PDGFR-α, NRP2, OR14I1, CD147 and CD46) [2,6]. Subsequent gB activation facilitates the fusion of the viral envelope with the cellular membrane, delivering the viral nucleocapsid to the cytoplasm. Translocation to the nucleus allows for viral DNA release and initiation of a sequential gene expression cascade categorized into immediate-early (IE), early (E) and late (L) phases. The major IE genes (MIE) UL123 and UL122 (IE1 and IE2) are the first expressed orchestrating the subsequent expression of early genes that, in collaboration with cellular factors, are critical for viral replication [7].

Similar to other herpesviruses, HCMV establishes lifelong infection, persisting latently within the host, periodically reactivating and facilitating efficient transmission [8]. Furthermore, HCMV exhibits the capability to replicate in a wide range of cell types, including epithelial, mucosal, smooth muscle, fibroblast, macrophage, dendritic, hepatocyte and endothelial cells, thereby enabling extensive dissemination within and between individuals [9].

HCMV also displays neurotropic properties, infecting various cells within the nervous system, including neural progenitor cells, astrocytes, microglia and neurons [10,13]. Congenital HCMV infection, while potentially affecting multiple fetal organs, is associated with irreversible damage within the nervous system, persisting throughout life [14,15]. Previous studies have shown a potential correlation between the severity of neuropathological alterations and clinical outcomes depending on the developmental stage of the central nervous system (CNS) at the time of congenital infection [16,17]; however, the precise mechanisms underlying HCMV-induced neuropathogenesis during CNS development remain poorly understood.

Microglial cells are the primary immune cells of the CNS and play a multifaceted role in its maintenance and defence [18]. Microglia not only limit damage from infections and pathogen invasions but they are also vital in repairing neurons following injuries, whether from physical trauma or diseases such as Alzheimer’s disease (AD), Parkinson’s disease and multiple sclerosis [19]. A recent study has reported the presence of HCMV in the colon and microglia within the postmortem brain tissue of AD patients [20], but the mechanism by which HCMV and microglia interact is not well known. To define the impact of HCMV infection on microglia, we have characterized HCMV infection in a human C20 microglia cell line that maintains microglial morphology, presents multiple cell surface microglial markers and expresses proinflammatory cytokines following stimulation with tumour necrosis factor-alpha (TNF-α) [21]. We define the conditions for HCMV infection of C20 cells, as well as the efficacy of antiviral agents in infection prevention. These findings suggest the feasibility of utilizing C20 cells as a model for investigating the impact of HCMV infection in microglia.

## Methods

### Cell lines, antibodies, viruses and chemicals

The human C20 microglia cell line [21], HMC3 microglia cell line (ATCC®CRL-3304) and normal human neonatal dermal fibroblasts (NHDF, Lonza, CC-2509) were cultured in Dulbecco's Modified Eagle Medium (DMEM) (Corning, 10–013-CV). The ARPE-19 human retinal epithelial cells (ATCC, CRL-2302) were cultured in the DMEM and F-12 medium (Gibco, 11765–054) mixed at 1 : 1 ratio. Spontaneously arising retinal pigment epithelia (ARPE)-19 and Normal Adult Human Dermal Fibroblasts (NHDF) mediums were supplemented with 10% fetal bovine serum (FBS), 1 mM HEPES (Corning, 25–060 CI), 100 U ml^−1^ of penicillin and 100 g ml^−1^ of streptomycin (100X Pen/Strep, Corning, 30–002 CI), while the microglial medium was supplemented with 10% FBS and Pen/Strep but not with HEPES. All cell lines were kept at 37 °C with 5% CO_2_.

HCMV viruses AD169^R^ (derived from AD169 (BADrUL131-C4)) [22], AD169^IE2/YFP^ and TB40/E were propagated in NHDF fibroblast cells as described [23].

### Virus infectivity assay

The human C20 and HMC3 microglia were plated at 10,000 cells per well the day before infection with AD169^R^, AD169^IE2/YFP^ or TB40/E (varying m.o.i.) and incubated overnight at 37 °C, 5% CO_2_. HCMV infection was analysed at diffrent hours post-infection (h.p.i.) as described [23]. All samples were tested twice in technical replicates of three.

### Virus inhibition assay

HCMV was pre-incubated with monoclonal antibodies (mAbs) (starting dilution of 20 µg ml^−1^ antibody, 2X) using 3-fold dilutions to achieve a final range of 0.04–10 µg ml^−1^ antibody, NRP-2 (0.05–0.15 µg ml^−1^), heparin (50 µg ml^−1^) and N-arylpyrimidinamine (NAPA) compounds (starting dilution of 20 µM) containing virus (at indicated m.o.i.). Both were incubated at 37 °C for 1 h before the inoculum was added to microglia at 10,000 cells per well and incubated overnight at 37 °C, 5 % CO_2_. At 24 h.p.i., plates were analysed using the Celigo imaging cytometer as described above. All samples were tested twice in technical replicates of three.

### Statistical analysis

All statistical tests were performed using GraphPad Prism 8 software (La Jolla, CA). An asterisk identifies statistical significance as determined by the two-way ANOVA test and is denoted as *, *P*<0.05; **, *P*<0.01; ***, *P*<0.001 and ****, *P*<0.0001. The half maximal inhibitory concentration (IC_50_) values for each mAb were calculated using the four-parameter non-linear regression analysis using GraphPad Prism 8 once the concentrations had been transformed to a log scale. Error bars represent standard deviations.

## Results

### HCMV strains infect microglial cells

**Fig. 1.**
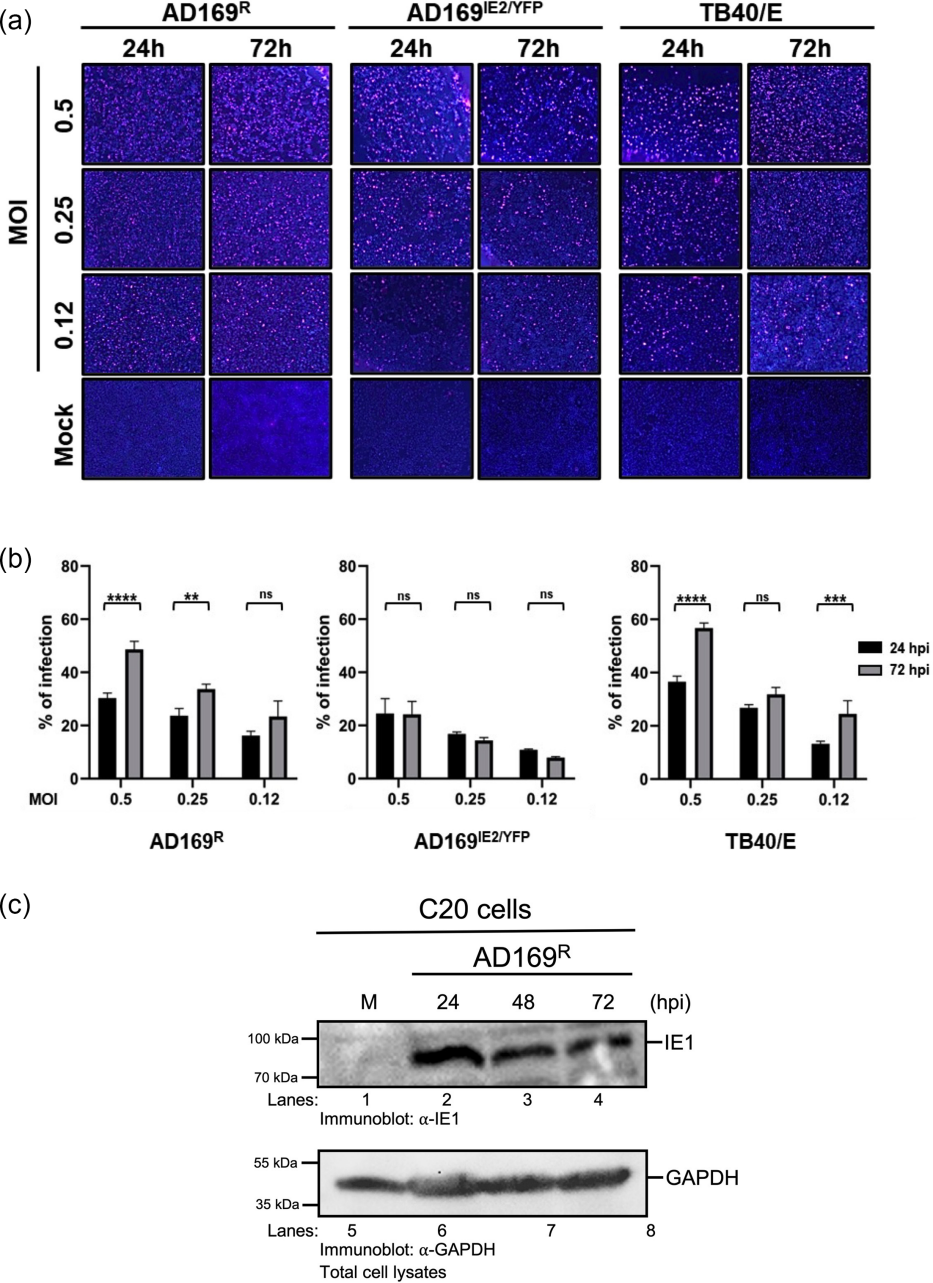
Human C20 microglia cells are permissive for HCMV infection. (**a**) Representative whole well of HCMV-infected C20 cells. Cells were infected with HCMV strains AD169^R^, AD169^IE2/YFP^ or TB40/E and incubated at 37 °C, 5% CO_2_. After incubation, cells were fixed with 4% paraformaldehyde, permeabilised with 0.3% Triton X-100 and stained with anti-IE1 antibody, followed by Alexa647-conjugated secondary antibody and Hoechst 33 342. Imaging was performed using the Celigo imaging cytometer. (**b**) The percentage of infection was calculated using non-infected cells as the 0% infection baseline. All conditions were performed in technical triplicate. Error bars represent standard deviation from the mean. Statistical significance is denoted as not significant (ns), **P<0.01; ***P<0.001; ****P<0.0001. (**c**) Total cell lysates from C20 cells infected with AD169^R^ (m.o.i. 0.1) were subjected to Western blot analysis using a rabbit anti-IE1 antibody and an anti-GAPDH antibody as a loading control. The molecular weight markers and immunoreactive proteins are indicated.

The human C20 microglia cell line was utilized as a proxy for the characterization of HCMV infection studies in microglia. The C20 cells were derived from human adult microglia and demonstrated to express microglia-specific factors and induce an inflammatory response [24,25]. Further, C20 cells were previously utilized to evaluate the effect of human immunodeficiency virus infection on the inflammatory response of microglia [21]. To determine the infectivity of HCMV on human C20 microglia, the infection of strains AD169^R^, AD169^IE2/YFP^ and TB40/E was evaluated for up to 72 h (Fig. 1). The AD169 laboratory strain has a 15 kb DNA segment deletion within the UL/b' region. Mutations at the UL128 locus are responsible for the lack of a pentamer, causing a loss of epithelial tropism [26]. This tropism was later restored by cloning the UL128/UL130/UL131A genes, generating a functional pentamer complex, resulting in the AD169^R^ strain [22]. Notably, strain TB40/E is a low-passage strain expressing both trimer and pentamer with wide tropism for epithelial cells, endothelial cells and macrophages [27].

HCMV strains were infected at m.o.i. of 0.12, 0.25 and 0.5 and examined for infection by staining for the expression of the HCMV IE-1 protein (Fig. 1). The microglia were susceptible to infection of viral strains, exhibiting detectable infection from 24 h.p.i across all m.o.i. as compared to the mock-infected cells (Fig. 1a). Furthermore, the overall infection levels were determined based on the total number of cells to assess virus infectivity among different strains and were found to increase at 72 h.p.i. As illustrated in Fig. 1b, microglia showed similar infection levels at 24 h.p.i. for all strains. However, at 72 h.p.i., the AD169^IE2/YFP^ strain did not exhibit an increase in infection, whereas the AD169^R^ and TB40/E strains showed increments of 49% and 53%, respectively. In summary, microglia are permissive for infection of clinical and laboratory strains of HCMV, with the TB40/E strain demonstrating the highest infectivity compared to AD169^R^ and AD169^IE2/YFP^ strains. Remarkably, the microglia were infectable with the laboratory strain AD169^IE2/YFP^ lacking a pentamer structure, suggesting that HCMV can infect microglia using the viral gH/gL/gO trimer; yet, the trimer may be insufficient for viral spread. These infection data were supported by demonstrating that the human HMC3 microglial cell line [28] was permissive for HCMV (Fig. S1). Furthermore, viral protein expression was validated by an immunoblot for IE1 (Fig. 1c) and late gene pp65 (Fig. S2A), supporting HCMV infection of C20 cells. Importantly, C20 cells could support virus replication upon evaluation of infectious virions generated from C20-infected cells (Fig. S2B). Collectively, these data demonstrate that C20 cells support virus infection and production.

### Receptor usage of HCMV infection of microglia

Upon establishing the ability of HCMV to infect microglia and induce expression of viral genes (Fig. 1), we conducted a study to elucidate the entry specificity of the virus in microglia. The trimeric complex appears to be implicated in microglial cell entry, evidenced by the ability of the AD169^IE2/YFP^ strain to infect microglia (Figs 1 and S1). However, infection levels were enhanced when the AD169^R^ strain, harbouring the restored pentameric complex, was employed. Consequently, we aimed to elucidate the role of the pentameric complex in infection and explore potential alternative mechanisms for microglial entry.

**Fig. 2.**
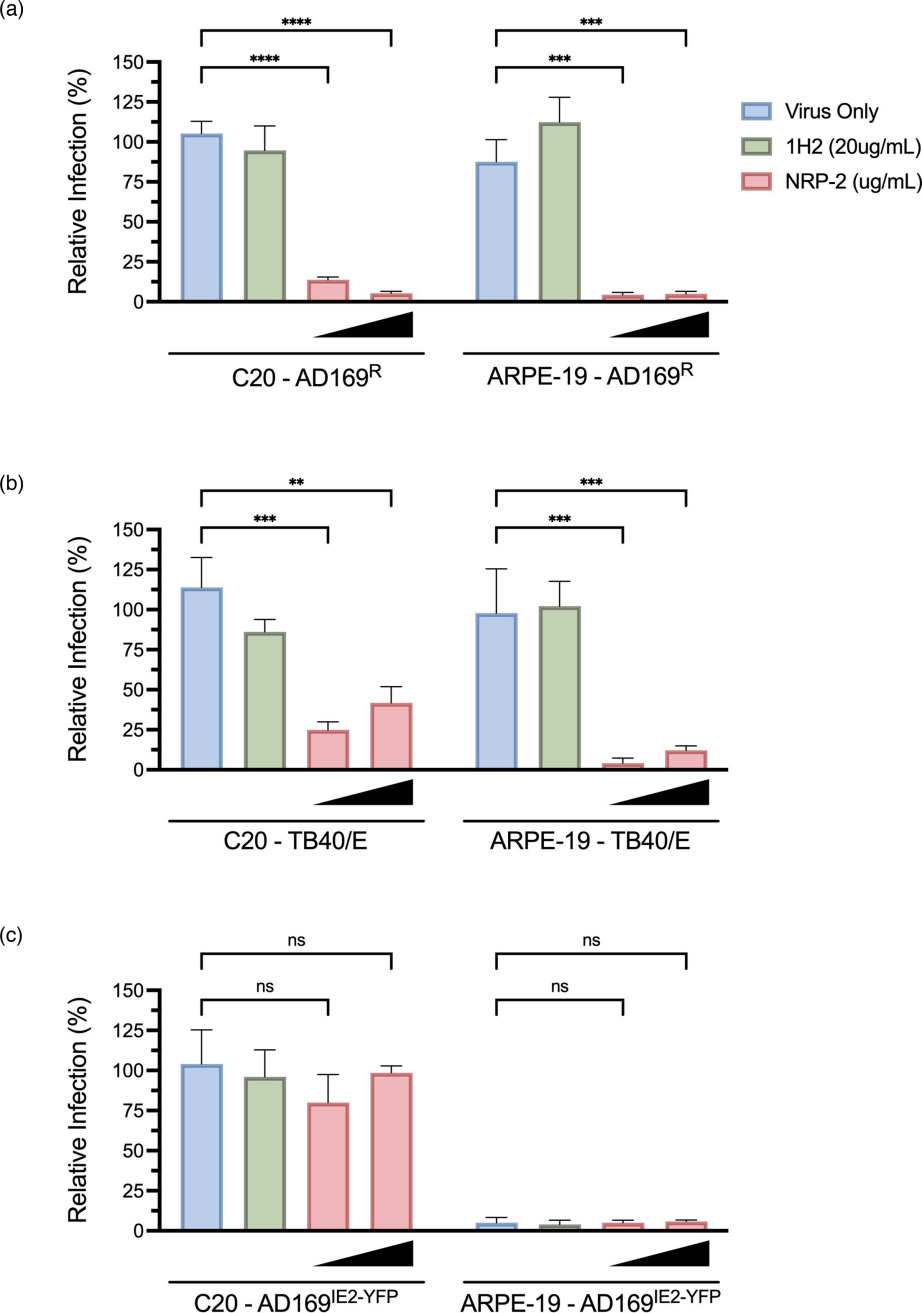
NRP-2 protein efficiently inhibits the entry of AD169^R^ into microglia. C20 microglia and ARPE-19 epithelial cells were infected with AD169^R^ (**a**), TB40/E (**b**) and AD169^IE2/YFP^ (**c**)(m.o.i. 0.5) pre-incubated with soluble NRP-2 (SinoBiological, 10695-H02H, 0.05–0.15 µg ml^−1^). A non-neutralizing antibody, 1H2 (20 µg ml^−1^), served as a negative inhibition control. Infection levels were assessed 24 h.p.i. Relative percent infection at 24 h.p.i. was determined following fixation and indirect immunofluorescence (IF) staining for IE1. All conditions were performed in technical triplicate. Error bars represent standard deviation from the mean. Statistical significance is denoted as **P<0.01; ***P<0.001; ****P<0.0001; ns, not significant.

To determine the role of the HCMV pentameric complex for entry into microglia, AD169^R^, AD169^IE2/YFP^ and TB40/E (m.o.i. 0.5) were pre-incubated with soluble NRP-2 (0.05–0.15 µg ml^−1^), along with non-specific antibody 1H2 (20 µg ml^−1^) as a negative inhibition control and analysed for infection 24 h.p.i. in ARPE-19 and C20 cells. ARPE-19 cells were used as experimental control for NRP-2-mediated inhibition. The AD169^R^ and TB40/E strains were significantly inhibited when incubated with NRP-2 protein in both C20 microglia and ARPE-19 cells (Fig. 2a, b). As expected, the laboratory strain AD169^IE2-YFP^ lacking a gH/gL pentamer that can use NRP-2 for entry was not affected by pre-incubation with NRP-2 (Fig. 2c). These findings suggest that the HCMV pentamer plays a role in microglia infection; yet, the trimer is sufficient for virus entry. Collectively, these findings suggest that HCMV entry can occur using different pathways in a single-cell type.

### Inhibition of HCMV into microglia by broadly neutralizing antibodies

**Fig. 3.**
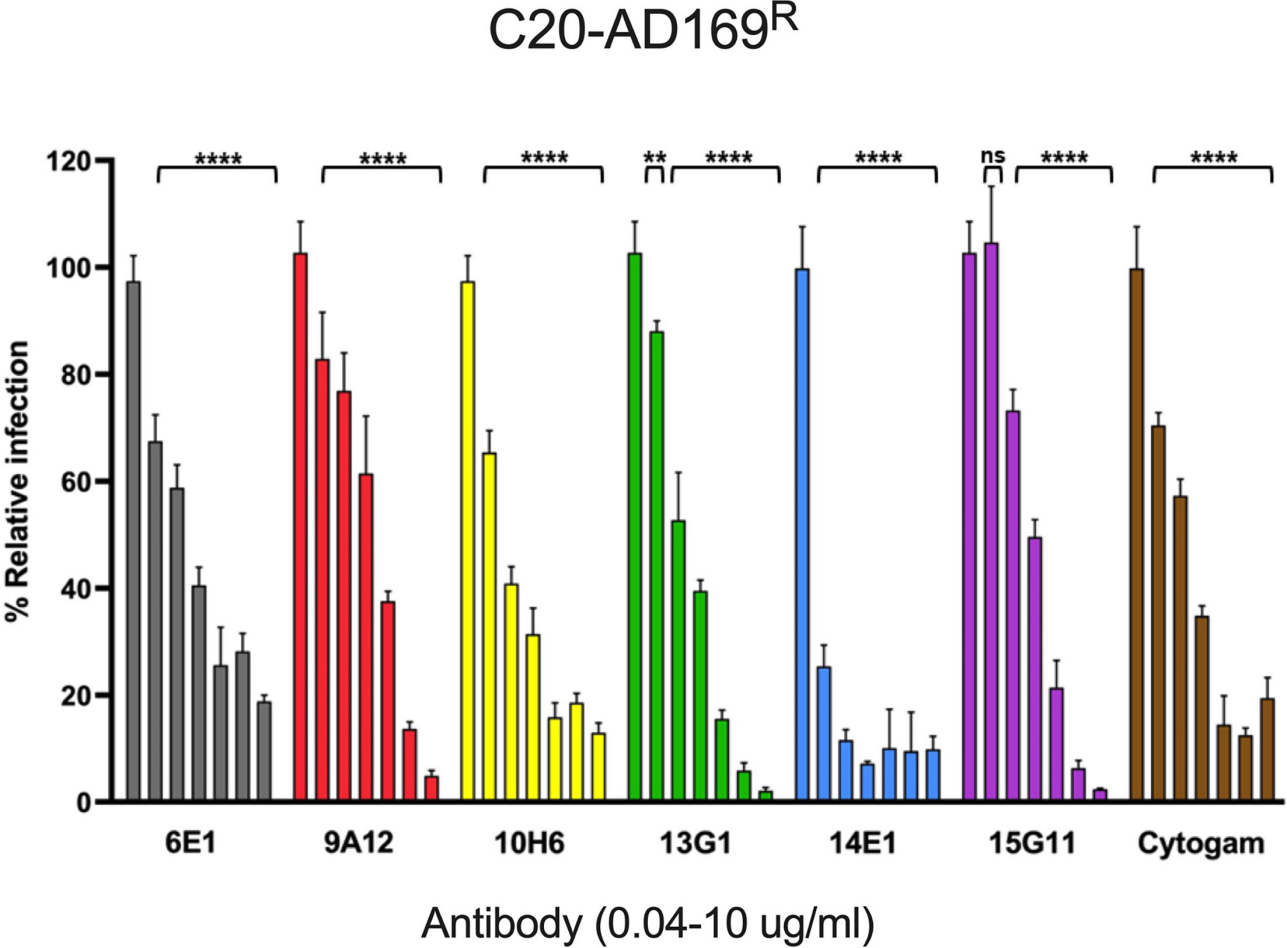
Anti-gH mAbs are broadly neutralizing HCMV in C20 cells. The mAbs and Cytogam (0.04–10 µg ml^−1^) were incubated with AD169^R^ (m.o.i. 0.2) to assess neutralizing capacity in C20 cells. Antibodies were diluted from 10 µg ml^−1^ using threefold dilution. Relative infection (%) at 24 h.p.i. was determined following fixation and indirect immunofluorescence (IF) staining for IE1. All conditions were performed in technical triplicate. Error bars represent standard deviation from the mean. Statistical significance is denoted as no significance (ns); **P<0.01; ****P<0.0001 based on the untreated sample, respectively.

We next evaluated whether HCMV-neutralizing antibodies against gH can effectively limit viral infection of microglia. The mAbs previously isolated by the group as proven to target the gH/gL complexes and broadly neutralize across various cell types were examined alongside HCMV strains in a neutralization assay in microglia cells [23,29] (Fig. 3). AD169^R^ (m.o.i. 0.2) was pre-incubated with respective mAbs (0–10 µg ml^−1^) prior to infection of microglia. All mAbs demonstrated high levels of protection in neutralization assays (>70%) 10 µg ml^−1^, indicating a substantial impact on virus infection. Among all antibodies, 14E1 emerged as the most potent showing an inhibition exceeding 70% against the AD169^R^ strain at the lowest tested concentration (0.04 µg ml^−1^). The half-maximal inhibitory concentrations (IC_50_ (µg/mL), Table 1) for the antibodies demonstrated significant limitation of infection (IC_50_ : 0.02–0.86 µg ml^−1^ using four-parameter non-linear regression analysis). Furthermore, the Food and Drug Administration (FDA)-approved HIG CytoGam® also exhibited neutralizing capacity in all tested concentrations. The effectiveness of the mAbs to inhibit viral infection in microglia cells follows a similar trend to the inhibition of epithelial cell infection, except for 10H6 and 14E1, which show a lower EC50 value in microglia cells. These mAbs have demonstrated varied inhibition with regard to viral strains and cell types, suggesting that the role of gH/gL complexes in virus entry may be different among cell types and viral strains. Despite these differences, the anti-gH/gL mAbs effectively neutralized HCMV infection in microglia, indicating that the gH protein could be a potential candidate for future therapeutics.

**Table 1.** Half-maximal inhibitory concentration (IC_50_) for anti-gH mAbs

Antibody	IC_50_ (µg ml^−1^)
6E1	0.13
9A12	0.86
10H6	0.08
13G1	0.19
14E1	0.02
15G11	0.35
Cytogam	0.15

### Evaluation of NAPA compounds as effective inhibitors of HCMV infection in microglia

We further sought to evaluate whether a chemical entry inhibitor could block HCMV infection of microglia. To this end, we used NAPA compounds referred to as MBX-4336 and MBX-4992 [30]. These compounds are effective entry inhibitors of viral infection and proliferation in fibroblasts and epithelial cells [31]. The compounds were demonstrated to block at an early stage of infection, persisting to the post-entry process for up to ~90 min following post-attachment [30,31].

**Fig. 4.**
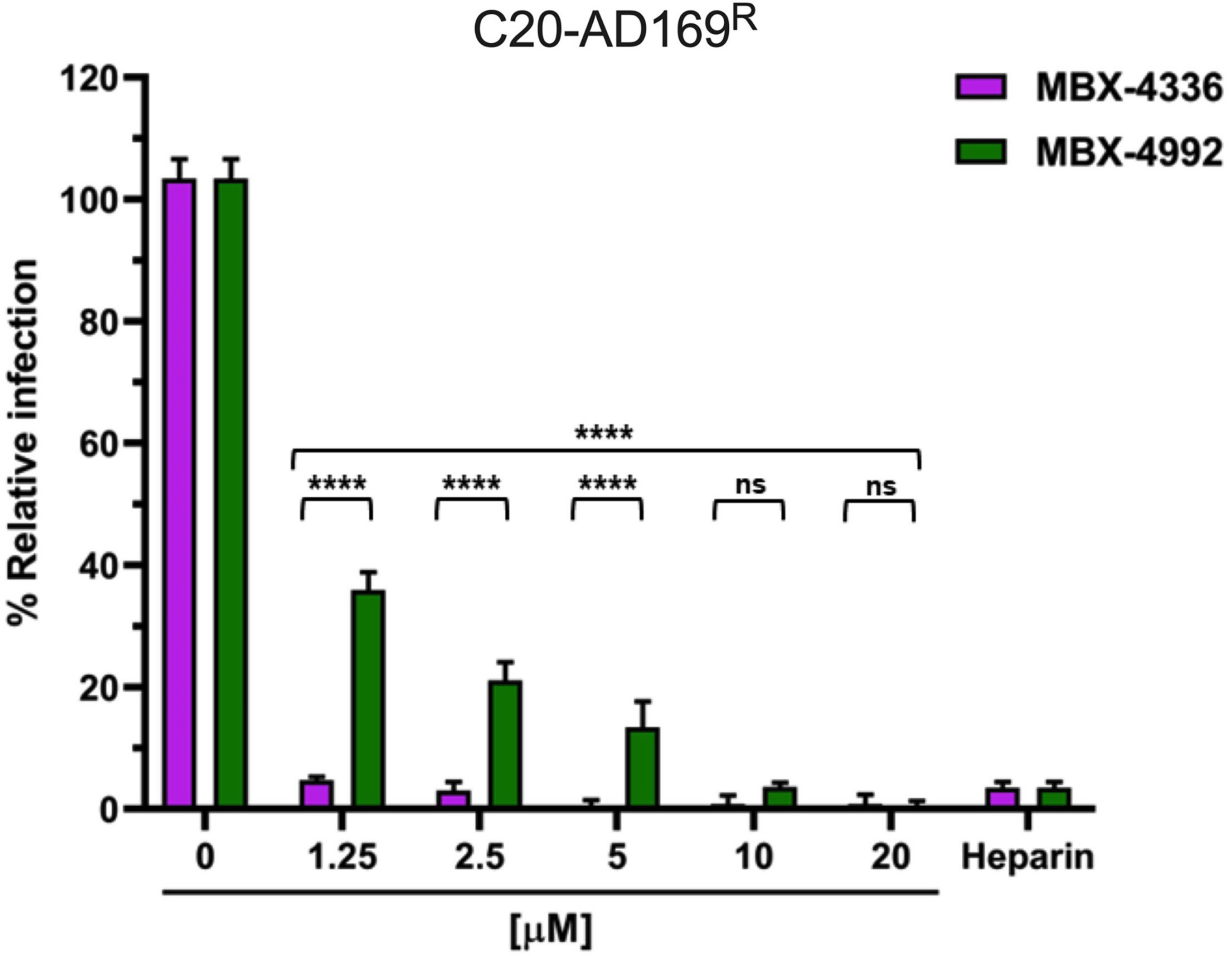
NAPA compounds MBX-4336 and MBX-4992 limit HCMV infection. C20 cells (10,000 cells per well) were incubated overnight with heparin (50 µg ml^−1^) and NAPA compounds (0–20 µM) along with AD169^R^ (m.o.i. 0.2). Relative infection (%) at 24 h.p.i. was determined following fixation and indirect immunofluorescence (IF) staining for IE1. All conditions were performed in technical triplicate. Error bars represent standard deviation from the mean. Statistical significance is denoted as non-significant (ns); ****P<0.0001 based on the untreated sample, respectively.

To evaluate the potential of these compounds in blocking HCMV infection, C20 microglia cells were treated with MBX-4336 and MBX-4992 (1.25, 2.5, 5, 10, 20 µM) followed by infection with AD169^R^ (m.o.i. 0.2). Heparin treatment (50 µg ml^−1^) was used as a positive control for blocking infection. The cells were evaluated for infection at 24 h.p.i. using an immunostain for IE1 (Fig. 4). Both compounds effectively inhibited HCMV infection by >95% at the highest tested concentration (20 µM). Additionally, compound MBX-4336 exhibited enhanced inhibitory capabilities at lower concentrations, inhibiting >90% at concentration 1.25 µM (Fig. 4). Based on published findings in fibroblast and epithelial cells, these compounds are more effective at limiting HCMV infection in C20 cells, with MBX-4336 blocking infection at higher levels than MBX-4992 [30,31]. Thus, the entry mechanism of HCMV in C20 cells may be more sensitive to the MBX-4336 and MBX-4992 entry inhibitors.

## Discussion

Herpesviruses, a diverse family of DNA viruses, are recognized for their capacity to induce a spectrum of human diseases, including significant neurological disorders [32]. Notable members of the *Herpesviridae* family implicated in neurological complications include herpes simplex virus type 1, known for causing herpes simplex encephalitis, and varicella-zoster virus, responsible for conditions such as postherpetic neuralgia and encephalitis upon reactivation [33]. Additionally, HCMV and Epstein–Barr viruses are associated with encephalitis and meningitis, overall illustrating the diverse neurological impact of herpesviruses [34].

Understanding the pathogenesis of HCMV-related neurological disorders is crucial for the development of effective therapeutic interventions. Research indicates that HCMV can directly infect neural cells, causing inflammation and tissue damage in the CNS [35,36]. Moreover, HCMV can disrupt immune responses, exacerbating neurological complications. Studies on congenital CMV highlight its potential to induce significant neurological deficits in newborns, including sensorineural hearing loss, cognitive and motor impairments, chorioretinitis and disseminated infection affecting multiple organs [14]. Recent investigations by Piccirilli *et al*. [17] have demonstrated HCMV’s tropism for neural stem/progenitor cells and neuronal committed cells in specific brain regions of infected human fetuses. This suggests a preferential targeting of immature neural cells, potentially contributing to structural and functional brain abnormalities associated with congenital HCMV infection [17].

The primary objective of this study was to establish conditions for viral infection of microglia to define the tropism of HCMV on brain-derived immune cells. Understanding these interactions is important for comprehending the neuropathogenesis of HCMV, particularly in congenital infections. Infection of C20 microglia by clinical and laboratory strains of HCMV suggests that these cells may serve as a valuable model for investigating viral neuronal tropism of HCMV. Notably, the data suggest that the viral pentamer complex likely plays a role in the entry of microglia, with inhibition of infection through blocking the NRP-2 receptor and mAbs targeting gH/gL complexes, completely underscoring the importance of these targets for viral entry (Figs2  3). Furthermore, an additional inhibition of HCMV by NAPA compounds MBX-4336 and MBX-4992 implies that viral infection occurs through common pathways among cell types.

*In vitro* models of neurons are valuable tools for studying neuronal infection by HCMV, enabling investigation into viral entry mechanisms, replication and disease pathogenesis [36,37]. Additionally, they can be utilized to assess the efficacy of novel antivirals and therapeutic strategies. Presently, the treatment of HCMV infection relies on antivirals such as ganciclovir, valganciclovir and foscarnet. While these drugs are effective at inhibiting viral replication, they may exhibit considerable side effects. The development of new antivirals with increased efficacy and reduced toxicity is an active area of research [38,40].

The susceptibility of microglia to HCMV infection raises important questions regarding the role of these cells in responding to viral invasion within the CNS. Microglia, as primary immune cells of the CNS [41,42], likely play a critical role in the immune response to HCMV. Upon activation by infection, microglia can contribute to inflammatory processes aimed at containing viral spread and clearing infected cells and debris. The immune response, however, may also lead to the release of inflammatory cytokines and neurotoxic substances, potentially exacerbating tissue damage [43].

The TB40/E strain displays efficient replication and a wide cell tropism, making it particularly suitable for studying HCMV infection in endothelial and monocyte-derived cells [27]. Our study demonstrated that microglia are permissive to infection by both clinical and laboratory strains of HCMV, with TB40/E exhibiting the highest infectivity compared to AD169^R^ and AD169^IE2/YFP^ strains. Remarkably, microglia were susceptible to infection even by the laboratory strain AD169^IE2/YFP^ lacking a pentameric structure, suggesting that HCMV can infect microglia using the virus trimer. These findings highlight the complexity of HCMV infectivity and stress the importance of utilizing clinically relevant virus strains in research.

The role of HCMV in neurodegenerative diseases like Alzheimer’s and Parkinson’s is an intriguing topic, evidenced by HCMV protein presence in the microglia of the brains of Alzheimer’s patients [44]. A possible scenario would be that HCMV infection of microglia may trigger an inflammatory response, increasing the risk of dementia and chronic inflammation associated with neurodegenerative diseases [45,46]. Moreover, HCMV has also been linked to other neurological disorders, including autism spectrum disorder, Huntington’s disease and Bell’s palsy [46]. Nevertheless, the relationship between HCMV and neurological disorders is not fully understood at the cellular level, and further research is necessary to clarify the role of HCMV in these conditions [46].

In summary, our findings using human C20 microglia suggest this cell type’s potential as a model for studying HCMV neurotropism and developing novel antiviral strategies targeting key viral entry mechanisms. Investigations into clinically relevant viral strains also provide insight into HCMV infectivity dynamics in microglia, essential for developing therapies for HCMV-associated neurological disorders. Further research is necessary to fully understand the complex interactions between HCMV and neurons and to improve clinical management of HCMV-related neurological complications.

## Supplementary material

10.1099/jgv.0.002096 Uncited Supplementary Material 1.

## Abbreviations

ARPE-19: Spontaneously arising retinal pigment epithelia-19
CNS: central nervous system
DMEM: Dulbecco's Modified Eagle Medium
HCMV: human cytomegalovirus
hpi: hours post-infection
mAbs: monoclonal antibodies
NAPA: N-arylpyrimidinamine
NHDF: Normal Adult Human Dermal Fibroblasts
NRP-2: neuropilin-2
