# Supplementary material for: Characterization of human cytomegalovirus infection dynamics in human microglia
Source: J Gen Virol. 2025 Apr 29;106(4):002096. doi: 10.1099/jgv.0.002096 (PMC12041478; doi:10.1099/jgv.0.002096)
Supplement: Uncited Supplementary Material 1. [file jgv-106-02096-s001.pdf]

Supplemental Figure 1

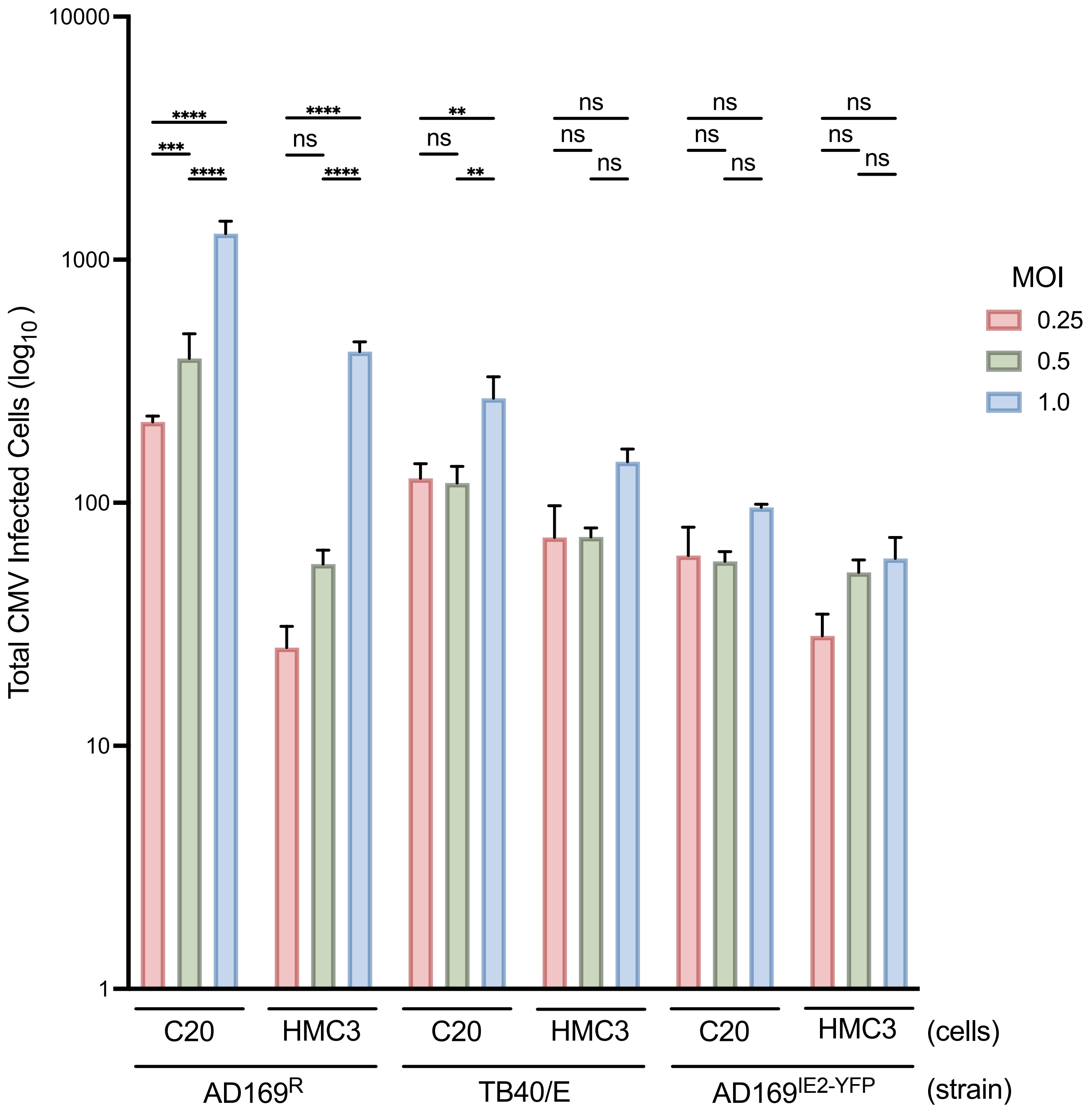

Supplemental Figure 1. HCMV infection of human microglia HMC3 cells. Human microglia C20 and HMC3 cells were infected with HCMV strains AD169^{R}, TB40/E, and AD169$^{IE2/YFP}$ for three days. Cells were fixed with 4% paraformaldehyde, permeabilized with 0.3% Triton X-100, and stained with anti-IE1 antibody, followed by Alexa647-conjugated secondary antibody and Hoechst. The number of HCMV infected cells were determined based on the IE1 stain using a Celigo Imaging Cytometer. All conditions were performed in technical triplicate. Error bars represent standard deviation from the mean. Statistical significance is denoted as non-significant (ns), **p<0.01; ***p<0.001; ****p<0.0001; ns, non-significant.

Supplemental Figure 2

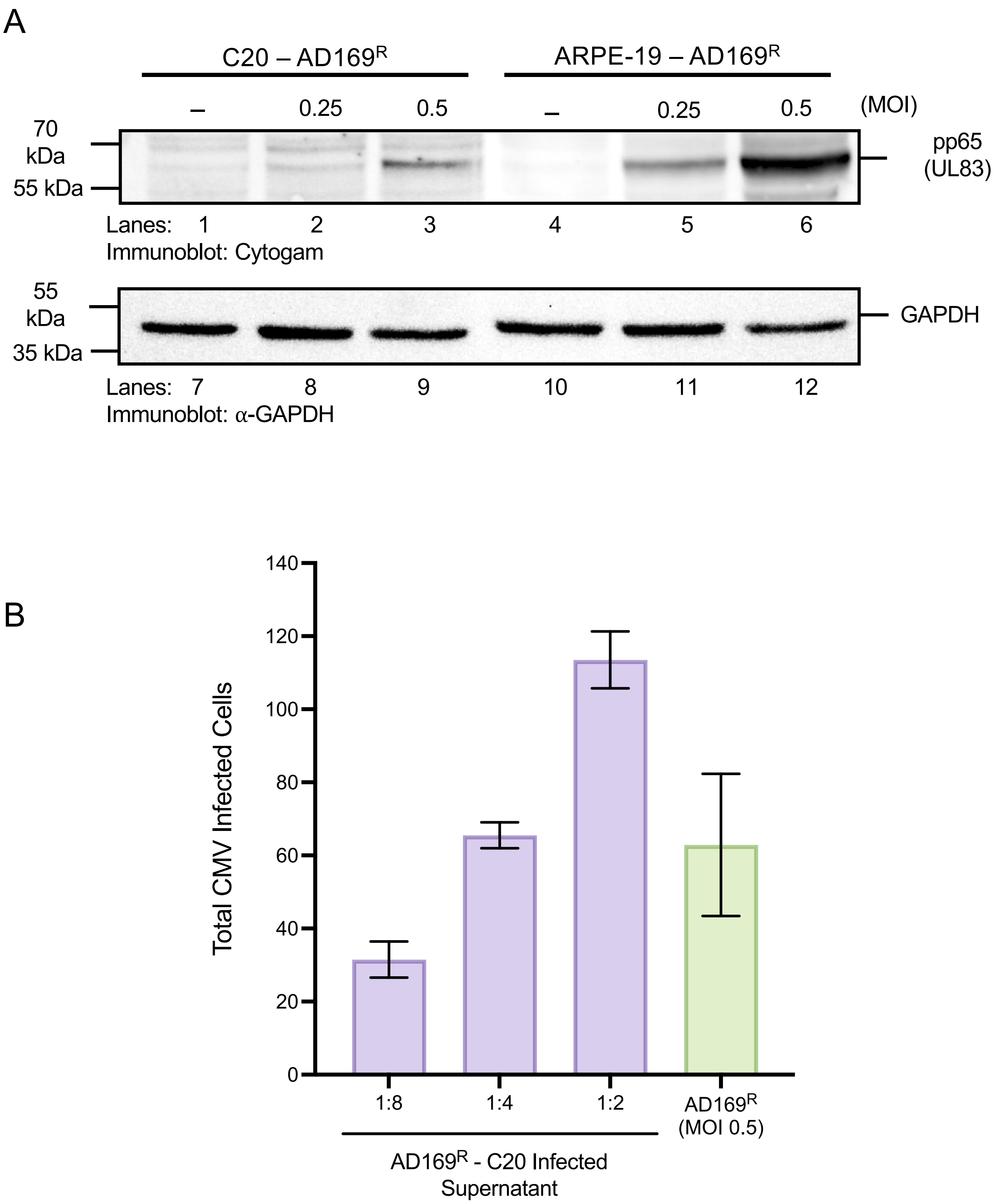

Supplemental Figure 2. Analysis of C20 infected cells and infectious particles. (**A**) Total cell lysates from C20 and ARPE-190 cells uninfected (-) or infected with AD169R (MOI 0.25 and 0.5) harvested 3 days post infection were subjected to Western blot analysis using CMV hyperimmunoglobulin Cytogam (Lanes 1-6) and an anti-GAPDH antibody (Lanes 7-12) as a loading control. The molecular weight markers and immunoreactive proteins are indicated.

(**B**) The supernatant from AD169^R-infected C20 were collected at 3 days post-infection and added to NDHF cells at varying ratios with complete media (1:8, 1:4, and 1:2). As a positive control, NHDF cells were infected with purified AD169^R (MOI:0.5). Cells were fixed with 4% paraformaldehyde, permeabilized with 0.3% Triton X-100, and stained with anti-IE1 antibody, followed by Alexa647-conjugated secondary antibody and Hoechst. The number of HCMV infected cells were determined based on the IE1 stain using a Celigo Imaging Cytometer. All conditions were performed in technical triplicate. Error bars represent standard deviation from the mean.
